# Neutrophils in HIV pathogenesis: dual roles, clinical implications, and therapeutic frontiers

**DOI:** 10.1097/MS9.0000000000003474

**Published:** 2025-06-13

**Authors:** Emmanuel Ifeanyi Obeagu

**Affiliations:** Department of Biomedical and Laboratory Science, Africa University, Mutare, Zimbabwe

**Keywords:** chronic inflammation, human immunodeficiency virus, immune activation, neutrophil extracellular traps, viral dissemination

## Abstract

Neutrophils, the most abundant innate immune cells, play a complex role in human immunodeficiency virus (HIV) infection, balancing between protective immunity and pathogenic inflammation. Initially, neutrophils contribute to early viral containment through phagocytosis, reactive oxygen species (ROS) production, and neutrophil extracellular traps (NETs). However, their excessive activation in chronic HIV infection can lead to systemic inflammation, immune dysfunction, and tissue damage. Despite their significance, neutrophils remain underexplored in HIV research compared to CD4+ T cells and macrophages. This review highlights the dual nature of neutrophils in HIV pathogenesis, emphasizing their involvement in immune dysregulation, disease progression, and associated comorbidities such as cardiovascular and metabolic disorders. While NETs can entrap and neutralize HIV, their overproduction exacerbates endothelial dysfunction and inflammation. Additionally, HIV-induced neutrophil dysfunction impairs pathogen clearance, further compromising immune defense. The implications of this review extend to potential therapeutic interventions targeting neutrophil-mediated inflammation. Strategies such as NET inhibitors, antioxidants, and immune modulators could help balance neutrophil function, reducing HIV-related complications while preserving antimicrobial defense. Future research should focus on developing precision therapies that mitigate the detrimental effects of neutrophils without compromising their protective roles, ultimately improving the prognosis and quality of life for people living with HIV.

## Introduction

Neutrophils, the most abundant circulating leukocytes in humans, play a central role in the innate immune response, acting as first responders to infection through mechanisms such as phagocytosis, degranulation, and the release of neutrophil extracellular traps (NETs). These cells rapidly infiltrate sites of infection or tissue injury and mount responses aimed at containing pathogens and modulating the inflammatory environment. They also interact closely with other immune cells to bridge innate and adaptive immunity. Their tightly regulated function is crucial to maintaining immune homeostasis and preventing tissue damage from excessive inflammation^[1,2]^. Neutrophil function is orchestrated through several pathways, including the activation of nuclear factor kappa-light-chain-enhancer of activated B cells, reactive oxygen species (ROS) production, and granule enzyme release. NETosis, a distinct form of cell death characterized by the extrusion of chromatin fibers laced with antimicrobial proteins, represents an important antimicrobial strategy. However, under pathological conditions, dysregulation of these pathways may result in bystander tissue injury and the promotion of chronic inflammation. This balance between host defense and immunopathology is a defining feature of neutrophil biology^[3,4]^. In the context of human immunodeficiency virus (HIV) infection, there is growing recognition that neutrophils play roles beyond traditional antimicrobial defense. While HIV research has primarily focused on CD4⁺ T cells, macrophages, and dendritic cells, emerging evidence suggests that neutrophils are also significantly affected by HIV infection – both directly and indirectly. HIV-associated neutropenia, functional exhaustion, excessive NET formation, and altered trafficking patterns have all been reported, indicating a complex and multifaceted involvement of neutrophils in HIV pathogenesis^[5,6]^.

**HIGHLIGHTS**

- Dual role: neutrophils act as both defenders against human immunodeficiency virus (HIV) and contributors to chronic inflammation and immune dysfunction.
- Neutrophil extracellular traps (NETs) in HIV: NETs can trap HIV but also exacerbate inflammation and tissue damage.
- Dysfunction: HIV impairs neutrophil function, reducing their antimicrobial capacity while promoting apoptosis and immune exhaustion.
- Comorbidities: Neutrophil-driven inflammation contributes to cardiovascular, neurological, and metabolic complications in HIV-infected individuals.
- Therapeutic targeting: Modulating neutrophil activity through anti-inflammatory drugs, NET inhibitors, and immune boosters may improve HIV management.

Despite these findings, the specific mechanisms by which HIV alters neutrophil behavior remain inadequately understood. There is a clear gap in knowledge regarding whether HIV directly infects neutrophils, how it modifies neutrophil signaling cascades, and to what extent these alterations contribute to immune dysfunction and viral persistence. For instance, the contribution of neutrophil-mediated immune suppression, such as through granulocytic myeloid-derived suppressor cells (G-MDSCs), and their potential to promote immune exhaustion in chronic HIV infection remain unresolved^[7,8]^. Moreover, the interplay between chronic inflammation and neutrophil dysregulation has not been fully delineated. It is unclear whether neutrophil dysfunction is primarily a consequence of systemic immune activation or whether it plays a causal role in perpetuating this inflammatory state. Neutrophils may also influence the recruitment and activation of CD4⁺ T cells, creating a feedback loop that facilitates viral replication. However, the molecular mediators of this interaction remain largely speculative^[9,10]^. Adding to this complexity is the potential for neutrophils to serve as transient viral carriers or facilitators of viral dissemination. While their short lifespan has led many to discount their role as true HIV reservoirs, the hypothesis that they may contribute to viral spread through trans-infection or by harboring HIV in NETs requires further exploration. Recent studies have hinted at epigenetic modifications and altered receptor expression in neutrophils from HIV-infected individuals, but these findings are preliminary and need deeper mechanistic validation^[4,11]^. Thus, a critical knowledge gap remains regarding how HIV reprograms neutrophils at the molecular, cellular, and systemic levels. Uncovering the nuances of this interaction could reveal novel therapeutic targets for mitigating HIV-associated inflammation and immune dysfunction. To date, few comprehensive reviews have synthesized the emerging evidence of neutrophil involvement in HIV pathology or contextualized these findings within the broader framework of immune regulation[12]. This review aims to fill this gap by providing an updated, mechanistic overview of neutrophil responses in HIV infection. The paper highlights both protective and pathological aspects of neutrophil activity, delineate known and hypothesized interactions with the virus and the immune system, and explore the therapeutic implications of targeting neutrophil pathways. In doing so, the paper underscores the urgent need for more targeted research into the immunobiology of neutrophils in HIV, particularly in the era of antiretroviral therapy and immune restoration.

## Aim

The aim of this article is to provide a comprehensive review of the crucial role that neutrophils play in the pathogenesis and progression of HIV infection.

## Rationale

Understanding the role of neutrophils in HIV infection is pivotal for several reasons. Neutrophils, traditionally recognized for their rapid response to bacterial and fungal infections, have increasingly been implicated in the immune response against viruses, including HIV. Despite being predominantly involved in innate immunity, neutrophils exhibit nuanced interactions with HIV that extend beyond their traditional antimicrobial roles. This complexity warrants a thorough exploration to uncover their precise contributions to HIV pathogenesis. Neutrophils play a dual role in HIV infection, functioning both as defenders against viral particles through mechanisms such as NET formation and as contributors to chronic inflammation and immune dysregulation. These opposing roles underscore the need for a balanced immune response in HIV-infected individuals. Moreover, the study of neutrophils in HIV infection holds promise for broader implications in immunology and virology research. Neutrophils’ interactions with HIV provide insights into the intricate interplay between innate and a viral infections. By elucidating these interactions, researchers can potentially identify novel biomarkers of disease progression, develop innovative therapeutic strategies targeting neutrophil function, and refine approaches to vaccine development against HIV. Furthermore, addressing the role of neutrophils in HIV infection aligns with current efforts to advance personalized medicine approaches. Given the variability in neutrophil responses among individuals and across different stages of HIV infection, personalized therapeutic strategies could optimize treatment outcomes and improve the quality of life for HIV-infected individuals. This rationale underscores the importance of integrating insights from neutrophil research into comprehensive HIV management strategies tailored to individual patient needs.

## Review methods

This narrative review was conducted to comprehensively evaluate the evolving understanding of neutrophils in the context of HIV infection, with a focus on their dualistic roles in host defense and immune pathology. A structured literature search was performed across three major scientific databases: PubMed, Scopus, and Web of Science. The time frame for included studies spanned from January 2000 to April 2024, capturing both foundational research and the most recent advances in the field. To ensure comprehensive coverage, a combination of specific search terms was used, including: “neutrophils AND HIV,” “NETs AND AIDS,” “neutrophil extracellular traps AND HIV,” “granulocytic myeloid-derived suppressor cells (G-MDSCs) AND HIV,” “neutrophil dysfunction AND AIDS,” and “HIV AND neutrophil activation.” Boolean operators and Medical Subject Headings terms were applied when appropriate to refine results. Inclusion criteria were limited to original, peer-reviewed research articles and reviews published in English that focused on experimental, clinical, or translational studies examining the role of neutrophils or neutrophil subsets in the pathogenesis, immune response, or treatment of HIV/AIDS. Studies involving in vitro models, in vivo animal research, and human cohort studies were all considered if they provided insights into neutrophil behavior or function in the context of HIV. Exclusion criteria involved non-English publications, case reports without mechanistic insight, conference abstracts lacking peer review, and articles focusing on neutrophils in unrelated infectious diseases unless a clear HIV connection was established.

A basic quality assessment was carried out by evaluating the peer-reviewed status of the publication, the methodological clarity, the scientific rigor, and the relevance of the study objectives to the review topic. Preference was given to studies with clearly described methodologies, appropriate controls, and those contributing mechanistic or clinically relevant data. Where available, systematic reviews and meta-analyses were used to support overarching themes or synthesize findings. This methodological approach ensured the inclusion of high-quality, relevant literature to accurately reflect the current understanding of neutrophil roles in HIV immunity and to inform future research and therapeutic strategies.

## Neutrophil heterogeneity and G-MDSCs

Neutrophils, once considered a relatively uniform population of terminally differentiated cells, are now increasingly recognized for their functional heterogeneity, especially under chronic inflammatory conditions such as HIV infection. This spectrum of neutrophil subsets includes populations with distinct phenotypic and functional profiles, ranging from pro-inflammatory responders to immunosuppressive regulators. Among the most studied immunosuppressive neutrophil subsets are the G-MDSCs, which expand significantly in HIV and other chronic viral infections^[13,14]^. G-MDSCs are characterized by surface markers such as CD11b⁺, CD15⁺, and low or absent HLA-DR expression, and they arise as part of a dysregulated myelopoiesis in response to persistent immune activation. Unlike conventional neutrophils that typically promote pathogen clearance, G-MDSCs exert potent immunosuppressive effects through mechanisms including arginase-1 production, ROS release, and induction of inhibitory cytokines such as IL-10 and TGF-β. These mediators collectively impair T cell proliferation and survival, particularly affecting CD8⁺ cytotoxic T lymphocytes, which are central to controlling HIV replication^[15,16]^.

In HIV-infected individuals, elevated levels of G-MDSCs have been associated with CD8⁺ T cell dysfunction, marked by reduced effector activity, impaired proliferation, and an exhausted phenotype. These suppressive effects are further amplified by upregulation of immune checkpoint molecules such as PD-L1 on G-MDSCs, which interact with PD-1 on T cells to blunt antiviral responses. The expansion of G-MDSCs thus contributes to a hostile immune microenvironment, where the cytotoxic arm of the adaptive immune response is suppressed, allowing for viral persistence and immune escape^[17,18]^. Moreover, the persistence of G-MDSCs even in patients on antiretroviral therapy (ART) suggests that their suppressive functions may continue to dampen immune reconstitution, complicating efforts to achieve full viral control or immune recovery. Understanding neutrophil heterogeneity and the immunosuppressive role of G-MDSCs in HIV provides critical insight into the non-viral mechanisms of immune dysfunction, and highlights these cells as potential therapeutic targets to restore effective antiviral immunity^[16,19]^.

## Neutrophils and HIV entry

HIV is known for its selective targeting of CD4+ T cells, which play a central role in adaptive immune responses. However, recent research has revealed that HIV can interact with other immune cell types, including neutrophils. Neutrophils, typically associated with the innate immune system’s rapid response to infections, have been shown to play a role in HIV entry and infection. This interaction adds a layer of complexity to our understanding of how HIV invades the human body and may have implications for disease progression and potential therapeutic interventions^[13,14]^. Neutrophils are equipped with various receptors, including CD4, CCR5, and CXCR4, which are also the primary receptors and co-receptors for HIV entry into CD4+ T cells. Although neutrophils express these receptors at lower levels compared to CD4+ T cells, they are not immune to HIV’s influence. HIV can bind to neutrophils via these receptors, albeit with lower affinity. This interaction may facilitate the initial stages of HIV entry into the host^[15,16]^. One intriguing aspect of the neutrophil-HIV interaction is the potential for neutrophils to act as reservoirs for the virus. Neutrophils are short-lived cells, and the presence of HIV within them might not lead to long-term infection. However, research has shown that HIV captured by neutrophils can be transferred to CD4+ T cells, enhancing viral dissemination. This suggests that neutrophils may play a role in the initial establishment of infection and viral load amplification^[17–19]^.

Beyond acting as potential reservoirs, neutrophils may also be involved in HIV transmission. HIV transmission often occurs at mucosal surfaces, and neutrophils can be found in mucosal tissues. Research has suggested that neutrophils can facilitate HIV transmission through a process known as transcytosis. This inflammation can contribute to the recruitment of other immune cells to the site of infection, including CD4+ T cells, which are the primary targets for HIV. Neutrophil-mediated inflammation may enhance the likelihood of CD4+ T cell infection and contribute to the initial stages of HIV pathogenesis^[20,21]^. The interaction between neutrophils and HIV is a complex and multifaceted process. While neutrophils express lower levels of the receptors required for HIV entry compared to CD4+ T cells, their involvement in HIV infection cannot be overlooked^[22–24]^.

## How HIV alters neutrophil function: direct and indirect mechanisms

Neutrophils, while historically overlooked in the context of HIV research, are increasingly recognized as important participants in the virus’s complex immunopathology. Their functional impairments in HIV-infected individuals – ranging from altered chemotaxis and phagocytosis to exaggerated NET formation and apoptosis – raise key questions about whether these changes arise from direct viral interactions or result indirectly from systemic immune disturbances. Direct effects of HIV on neutrophils refer to instances where the virus or its components engage neutrophils through specific receptors or intracellular pathways. Although neutrophils are generally considered non-permissive to productive HIV replication due to their short lifespan and limited transcriptional machinery, several studies suggest that HIV can still interact with these cells at the surface or subcellular level. For instance, the binding of HIV envelope glycoproteins (gp120/gp41) to receptors such as CCR5, CXCR4, or even DC-SIGN-like molecules expressed on neutrophils can trigger aberrant signaling cascades. These interactions may induce premature activation, NETosis, or the release of pro-inflammatory cytokines, thereby modifying neutrophil function without actual infection. Moreover, emerging evidence suggests that HIV may induce epigenetic reprogramming in neutrophils, altering chromatin structure and transcription factor accessibility in ways that influence their phenotype, lifespan, and immune behavior – even in the absence of viral replication. Such modifications could contribute to long-term immune dysregulation in people living with HIV^[20,21]^.

Indirect effects, on the other hand, stem from the broader immunological milieu of HIV infection, particularly the chronic immune activation and inflammation that characterize untreated and even treated HIV. In this pro-inflammatory environment, circulating cytokines such as interleukin-6 (IL-6), tumor necrosis factor-alpha (TNF-α), and IFN-α influence neutrophil maturation and activation. This chronic stimulation may drive the emergence of dysfunctional neutrophil subsets, such as low-density granulocytes and G-MDSCs, which possess immunosuppressive capabilities and can exacerbate CD4⁺ T-cell exhaustion. Additionally, HIV-induced bone marrow dysfunction may impair granulopoiesis, contributing to neutropenia and the release of immature or dysregulated neutrophils into circulation. Antiretroviral therapy (ART), while essential for viral suppression, may not fully restore normal neutrophil function, particularly in individuals with persistent immune activation or co-infections such as tuberculosis (Table 1)^[22–24]^.

**Table 1:** Neutrophil dysfunctions and associated HIV-related comorbidities

Neutrophil dysfunction	Description	Associated comorbidities in HIV
Reduced phagocytosis	Impaired ingestion and clearance of pathogens	Increased susceptibility to opportunistic infections
Excessive ROS production	Overactivation of oxidative burst pathways	Oxidative stress, endothelial damage, accelerated aging
NET dysregulation	Excessive or persistent NET release	Vascular injury, thrombosis, autoimmune responses
Altered chemotaxis	Defective migration toward inflammatory signals	Delayed resolution of infection, tissue inflammation
Prolonged survival and delayed apoptosis	Increased lifespan of activated neutrophils	Chronic inflammation, tissue damage
Hyperactivation and cytokine release	Elevated secretion of IL-8, TNF-α, and other inflammatory mediators	Immune activation, cytokine storm, CD4+ T cell recruitment
Epigenetic reprogramming	Changes in gene expression affecting immune response	Persistent immune dysregulation, impaired ART responsiveness
Interaction with G-MDSCs	Expansion of immunosuppressive granulocytic populations	CD8+ T cell exhaustion, poor viral control

## Neutrophils as viral reservoirs

The idea of neutrophils acting as viral reservoirs in HIV infection has sparked considerable scientific curiosity and debate. Traditionally, viral reservoirs in HIV refer to long-lived cells such as resting memory CD4⁺ T cells and tissue-resident macrophages that harbor integrated, replication-competent HIV proviruses, allowing the virus to persist despite antiretroviral therapy (ART). Neutrophils, by contrast, are short-lived innate immune cells with a typical lifespan of less than 24 hours in circulation, making them unlikely candidates for serving as long-term viral reservoirs in the classical sense^[20,21]^. However, emerging evidence suggests that neutrophils may transiently internalize HIV virions, RNA fragments, or viral proteins, potentially through mechanisms such as phagocytosis or receptor-mediated interactions, including Fc receptor or complement receptor engagement. Some studies have detected HIV-related genetic material or antigens within neutrophils, raising the possibility that these cells may act as short-term carriers of viral components rather than as sites of latent infection or replication[22].

This transient carriage could still have important immunopathological consequences. For example, neutrophils may facilitate viral dissemination by trafficking through inflamed tissues and interacting with other immune cells, such as dendritic cells or CD4⁺ T cells, potentially enhancing the exposure of these target cells to the virus. Additionally, activated neutrophils can release NETs that may entrap HIV particles and immune complexes, contributing to inflammation and immune activation without effectively clearing the virus[23]. Moreover, although no definitive evidence confirms active HIV replication within neutrophils, their capacity to harbor viral components and modulate immune environments supports their role as transient intermediaries in HIV pathogenesis. Their interactions with the virus may amplify immune activation, shape inflammatory microenvironments, and possibly influence viral persistence indirectly, even if they do not constitute classical reservoirs themselves[24].

## Neutrophils in HIV transmission

Neutrophils are equipped with various antimicrobial mechanisms, including the release of ROS and antimicrobial peptides, making them essential components of mucosal immunity^[25–27]^. One of the ways in which neutrophils contribute to HIV transmission is through a process known as transcytosis. Transcytosis involves the transport of HIV particles across mucosal barriers by neutrophils. During transcytosis, neutrophils can capture HIV particles from the mucosal surface and transport them through the epithelial cells lining the mucosa to deliver the virus to underlying target cells, such as CD4+ T cells. This mechanism may provide a means for HIV to bypass the mucosal barrier and establish infection within the host^[28,29]^. Neutrophils are capable of capturing HIV and increasing the local viral load. This phenomenon has been observed in the genital mucosa, where neutrophils can capture and store HIV particles. When these neutrophils migrate to lymphoid tissues, they can release the virus and potentially transmit it to CD4+ T cells and other immune cells, amplifying the infection^[30,31]^. Neutrophils also contribute to the inflammatory response at mucosal sites following exposure to HIV. The inflammation triggered by neutrophils may attract other immune cells, including CD4+ T cells, to the site of infection. This recruitment of target cells, coupled with the presence of captured HIV in neutrophils, can enhance the likelihood of transmission and infection^[32,33]^. Strategies that modulate neutrophil-mediated inflammation or disrupt the transcytosis process may hold promise in reducing the risk of HIV transmission. Investigating the interplay between neutrophils and HIV transmission is essential for improving our overall understanding of how the virus spreads and for developing targeted interventions. Neutrophils, primarily known for their role in innate immunity, are intricately involved in HIV transmission[34].

## Neutrophils in HIV-associated inflammation

While much attention has been focused on the adaptive immune response and the role of CD4+ T cells, neutrophils have emerged as key players in HIV-associated inflammation. This paper delves into the multifaceted role of neutrophils in HIV-associated inflammation, shedding light on their contribution to the chronic inflammatory state seen in HIV-infected individuals[35]. Neutrophils are innate immune cells that rapidly respond to infections. In the context of HIV infection, they become activated in response to the presence of the virus. This activation results in the release of proinflammatory cytokines, including IL-6, TNF-α, and interleukin-8. These cytokines contribute to the overall inflammatory milieu in the host, triggering immune responses and promoting inflammation^[35]^. Neutrophils are equipped with the ability to recruit other immune cells to the site of infection. Their release of chemokines and cytokines can attract various immune cells, including CD4+ T cells. This recruitment fosters an environment in which immune cells are drawn to the sites of HIV replication and can become infected, ultimately contributing to viral spread[36].

One intriguing aspect of neutrophils in HIV-associated inflammation is their ability to release NETs. NETs are web-like structures composed of DNA, histones, and antimicrobial proteins. While they are designed to trap and neutralize pathogens, in the context of HIV infection, NETs may have unintended consequences. NETs have been associated with tissue damage and impaired CD4+ T cell function, which can exacerbate the inflammatory response and hinder the host’s ability to control viral replication[37]. HIV-induced immune activation is a hallmark of the infection, and this chronic inflammation is linked to disease progression and the development of non-AIDS-related comorbidities. Neutrophils, through their role in promoting inflammation and immune activation, may contribute to the pathogenesis of HIV and increase the risk of complications, such as cardiovascular disease and neurocognitive disorders[38]. Strategies aimed at modulating neutrophil responses, such as the inhibition of specific inflammatory pathways or NET formation, could be explored to mitigate the chronic inflammation associated with HIV infection. Targeting these processes may help improve patient outcomes and reduce the long-term complications of HIV infection. Neutrophils, once considered the first line of defense against infections, are actively involved in HIV-associated inflammation. Their activation, release of proinflammatory mediators, recruitment of immune cells, and the release of NETs contribute to the chronic inflammation seen in HIV-infected individuals[7].

## Neutrophils and immune dysfunction in HIV infection

This article explores the intricate relationship between neutrophils and immune dysfunction, revealing how these innate immune cells contribute to the overall immunological impairment observed in HIV-infected individuals[39]. Neutrophils are known for their ability to release NETs, web-like structures composed of DNA, histones, and antimicrobial proteins. In the context of HIV infection, NETs have been found to have both beneficial and detrimental effects. While NETs can trap and neutralize pathogens, they can also lead to immune dysfunction. The release of NETs has been associated with impaired CD4+ T cell function, hampering the adaptive immune response against HIV. As a result, NETs may inadvertently facilitate viral persistence and immune escape[40]. HIV infection is characterized by chronic immune activation and inflammation, even in the absence of opportunistic infections. Neutrophils, as prominent mediators of inflammation, contribute to this dysregulated immune response. The continuous activation and cytokine release by neutrophils can exacerbate the proinflammatory state, which, in turn, leads to immune exhaustion and dysfunction. Prolonged immune activation can exhaust CD4+ T cells and disrupt immune homeostasis^[41]^. Neutrophils can modulate immune responses through various mechanisms. Their interactions with other immune cells, such as dendritic cells and macrophages, may influence antigen presentation and cytokine production, the adaptive immune response. Neutrophil-mediated immune modulation can alter the host’s ability to mount effective immune responses against HIV, contributing to immune dysfunction^[42,43]^.

Neutrophils, through their interactions with chemokines and other signaling molecules can influence the trafficking of immune cells. This may impact the distribution of CD4+ T cells and other immune cells, affecting their localization in lymphoid tissues and mucosal sites where HIV replication is concentrated. Altered immune cell trafficking can disrupt immune surveillance and limit the body’s ability to combat the virus effectively[44]. Targeting specific pathways involved in neutrophil activation and function may help mitigate the chronic inflammation and immune exhaustion associated with HIV. Strategies to modulate NET release or inhibit the detrimental effects of neutrophil activation could improve the overall immune response and slow the progression of the disease. Neutrophils, typically considered the first responders to infections, play a nuanced role in immune dysfunction in HIV infection. Their involvement in the release of NETs, dysregulated inflammation, immune modulation, and influence on immune cell trafficking contributes to the overall immune impairment observed in HIV-infected individuals^[44,45]^.

## Neutropenia in HIV infection

Neutropenia, a condition characterized by abnormally low levels of neutrophils, is a common hematological complication in individuals with HIV. This decline in neutrophil count can occur at various stages of infection and is influenced by multiple factors, including direct viral effects, immune activation, opportunistic infections, and antiretroviral therapy (ART). In early HIV infection, transient neutropenia may result from immune system activation and redistribution of neutrophils to inflamed tissues. However, as the disease progresses, chronic neutropenia often develops due to bone marrow suppression, increased apoptosis, and the effects of inflammatory cytokines that impair neutrophil production and function^[40,41]^. The clinical implications of HIV-associated neutropenia are significant, as it increases susceptibility to bacterial and fungal infections, particularly in individuals with advanced immunosuppression. Opportunistic infections such as Staphylococcus aureus, Pseudomonas aeruginosa, and Candida species are more common in neutropenic HIV patients, contributing to prolonged hospitalizations and higher morbidity. Furthermore, severe neutropenia (absolute neutrophil count <500 cells/µL) can lead to life-threatening complications, including sepsis and poor wound healing. Antiretroviral medications, particularly zidovudine, lamivudine, and certain protease inhibitors, have been associated with bone marrow suppression, exacerbating neutropenia in some patients^[42,43]^. Management of HIV-related neutropenia involves addressing underlying causes, optimizing ART regimens, and using supportive therapies when necessary. In mild to moderate cases, neutropenia may not require intervention, but for severe cases, treatment options include granulocyte colony-stimulating factors to stimulate neutrophil production, adjusting ART regimens to minimize marrow toxicity, and proactive management of infections. Research continues to explore how modulating neutrophil function could reduce infection risk while minimizing the inflammatory complications associated with neutrophil activation^[44,45]^.

## Double-edged role of neutrophils in HIV immunity

**Figure 1.**
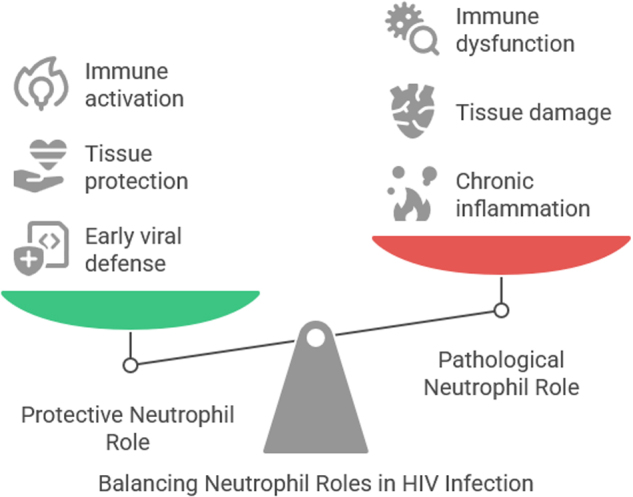
Neutrophil roles in HIV infection.

Neutrophils, as the most abundant circulating leukocytes and first responders in innate immunity, exhibit a double-edged role in the context of HIV infection. On the one hand, they contribute significantly to host defense through classical antimicrobial mechanisms, such as phagocytosis, production of ROS, and the formation of NETs – a process by which chromatin is expelled to entrap and neutralize pathogens, including HIV-infected cells. These functions underscore the protective side of neutrophils, helping to limit early viral dissemination and activate downstream immune pathways[33]. However, in chronic HIV infection, these same defensive strategies can become dysregulated, tipping the balance toward pathology. Persistent immune activation, a hallmark of untreated or advanced HIV, leads to excessive neutrophil activation, which in turn contributes to chronic inflammation and collateral tissue damage. Notably, NETs – though antimicrobial – can provoke endothelial injury, promote thrombosis, and perpetuate inflammatory loops that compromise mucosal barriers. Moreover, neutrophils secrete chemokines and cytokines that actively recruit CD4 + T cells – the primary targets of HIV – into sites of infection, inadvertently expanding the pool of susceptible cells and facilitating viral propagation[46]. This dual functionality highlights the complex immunobiology of neutrophils in HIV. While essential for acute defense, their sustained activation without resolution exacerbates immune dysfunction, contributes to comorbidities such as cardiovascular disease, and may impair long-term immune restoration despite antiretroviral therapy (ART). Therefore, therapeutic efforts must strike a careful balance – enhancing neutrophil antimicrobial functions where needed while suppressing their pro-inflammatory and tissue-damaging activities to avoid further immunopathology (Fig. 1)[47].

## Immune modulation

Immune modulation plays a critical role in the complex interplay between the HIV and the host immune system. This review examines the involvement of key cellular markers, namely CD80, CD83, CD86, PD-L1/PD1, and cytotoxic T-lymphocyte-associated protein 4 (CTLA-4), in shaping immune responses during HIV infection. Understanding these markers is essential for elucidating the mechanisms of immune dysfunction, chronic inflammation, and viral persistence observed in HIV-infected individuals[39]. CD80 (B7-1), CD83, and CD86 (B7-2) are co-stimulatory molecules expressed on antigen-presenting cells (APCs), including dendritic cells and macrophages. These molecules play a crucial role in T-cell activation and immune response regulation. During HIV infection, dysregulation of CD80, CD83, and CD86 expression can impair T-cell activation and contribute to immune dysfunction. HIV-mediated depletion of CD4+ T cells and chronic immune activation may alter the expression and function of these molecules, thereby affecting immune surveillance and response against the virus.[40] Programmed death-ligand 1 (PD-L1) and its receptor PD-1 are immune checkpoint proteins that regulate T cell responses and maintain immune tolerance. In HIV infection, upregulation of PD-L1 on infected cells and PD-1 on T cells contributes to T cell exhaustion and impaired immune function. The PD-L1/PD-1 axis is exploited by HIV to evade immune surveillance and promote viral persistence. Therapeutic strategies targeting this pathway, such as PD-1/PD-L1 checkpoint inhibitors, have shown promise in restoring T cell function and enhancing immune responses in HIV-infected individuals[41].

CTLA-4 is another immune checkpoint molecule that negatively regulates T-cell activation. CTLA-4 competes with CD28 for binding to CD80 and CD86 on APCs, resulting in inhibition of T-cell activation and proliferation. In HIV infection, dysregulation of CTLA-4 expression may contribute to immune dysfunction and impaired viral control. Strategies aimed at modulating CTLA-4 signaling could potentially enhance T cell responses and improve immune surveillance against HIV[42]. The dysregulation of these immune checkpoint underscores their potential as therapeutic targets. Novel immunotherapeutic approaches, such as immune checkpoint blockade and agonist therapies, hold promise for restoring immune function and enhancing viral control in HIV-infected individuals. However, challenges remain in balancing immune activation with potential risks of immune-related adverse events and viral rebound[43].

## HIV and dendritic cell-specific intercellular adhesion molecule-3-grabbing non-integrin

dendritic cell-specific intercellular adhesion molecule-3-grabbing non-integrin (DC-SIGN) is a C-type lectin receptor predominantly expressed on dendritic cells and certain macrophage subsets. It plays a well-documented role in enhancing HIV-1 transmission by binding to the viral envelope glycoprotein gp120, facilitating viral capture, and promoting trans-infection of CD4+ T cells. While this mechanism has been extensively studied in classical antigen-presenting cells, its relevance to neutrophils is increasingly gaining attention in the context of HIV pathogenesis[40]. Although mature neutrophils traditionally lack high levels of DC-SIGN expression under homeostatic conditions, emerging evidence suggests that inflammatory environments – such as those found in chronic HIV infection – can modulate neutrophil phenotype and function. Specifically, some neutrophil subsets may express DC-SIGN or functionally similar lectin receptors under pathological conditions. This opens a new avenue of inquiry into whether neutrophils can, under certain stimuli, interact with HIV in a manner similar to dendritic cells[41]. More importantly, even in the absence of direct DC-SIGN expression, neutrophils interact with DC-SIGN + dendritic cells in HIV-infected tissues. Through the release of cytokines and chemokines, neutrophils may indirectly enhance the ability of dendritic cells to capture and transmit HIV via DC-SIGN. Conversely, DC-SIGN-mediated viral uptake by dendritic cells can influence neutrophil behavior by promoting the secretion of inflammatory mediators that exacerbate neutrophil activation, NET release, and tissue inflammation[42]. Furthermore, the interactions between HIV, DC-SIGN + cells, and neutrophils contribute to the formation of immune synapses in lymphoid tissues, enhancing local viral replication and immune activation. This interconnected network supports a pathogenic loop where HIV-driven inflammation boosts neutrophil recruitment and activation, which in turn perpetuates the inflammatory milieu favorable for DC-SIGN-mediated viral propagation[43].

## Therapeutic interventions targeting neutrophil dysfunction in HIV

The evolving understanding of neutrophil involvement in HIV pathogenesis has opened new avenues for therapeutic intervention. Rather than being passive bystanders, neutrophils actively shape the immune environment through processes such as NETosis, cytokine release, and cellular crosstalk. However, when deregulated, these same mechanisms contribute to tissue damage, immune exhaustion, and persistent inflammation, even under antiretroviral therapy (ART). Addressing this neutrophil-mediated imbalance is becoming increasingly critical in the holistic management of HIV^[44,45]^. One promising approach is the targeted degradation of NETs, which are often exaggerated and misregulated in HIV-infected individuals. While NETs can ensnare pathogens, their chronic overproduction leads to endothelial damage, enhanced immune activation, and the recruitment of susceptible CD4⁺ T cells. DNase I, a recombinant enzyme that cleaves extracellular DNA, has shown potential in degrading these NETs and mitigating their pathological effects. Originally used in diseases like cystic fibrosis, DNase I is now being explored for its ability to curb neutrophil-driven inflammation in chronic infections, including HIV. Its utility may lie in reducing inflammatory burden and preserving vascular and mucosal integrity – key considerations in people living with HIV^[33,46–49]^.

In parallel, modulating neutrophil trafficking through chemokine receptor targeting has gained interest. Neutrophils are recruited to inflammatory sites through chemokine gradients, often amplified in HIV due to persistent immune activation. The CCR5 receptor, known for its role in HIV entry into T cells, is also expressed on neutrophils and governs their movement toward sites of inflammation. CCR5 inhibitors, such as maraviroc, have already been incorporated into ART regimens for their antiviral properties. However, their ability to alter neutrophil chemotaxis and tissue infiltration offers an additional immunomodulatory benefit. By attenuating neutrophil accumulation in vulnerable tissues, CCR5 inhibition may reduce collateral damage and immune activation, potentially aiding in mucosal healing and the preservation of barrier functions^[50–54]^. Furthermore, statins, widely known for their lipid-lowering effects, have shown immunomodulatory capabilities that extend to neutrophil-mediated inflammation. For example, atorvastatin has been reported to suppress neutrophil activation, reduce ROS production, and lower systemic inflammatory markers in HIV-infected individuals. Statins also appear to diminish the expression of adhesion molecules that facilitate neutrophil endothelial transmigration. When used as adjuncts to ART, these drugs may help dampen chronic inflammation without compromising host defense, thus improving cardiovascular outcomes and possibly ameliorating neuroinflammation – two common concerns in long-term HIV care^[55–62]^.

## Complementary therapies

Complementary therapies, particularly those involving antioxidants and micronutrients, have shown potential in supporting neutrophil function and overall immune health in individuals living with HIV. HIV infection is frequently accompanied by elevated oxidative stress and chronic inflammation, which impair neutrophil activity and contribute to immune dysregulation. Antioxidants such as vitamin C, vitamin E, and glutathione precursors help neutralize excess ROS, which are often overproduced in HIV and can lead to neutrophil dysfunction, tissue damage, and impaired microbial clearance. By modulating oxidative stress, these antioxidants may enhance neutrophil viability and function, especially in maintaining proper phagocytic capacity and limiting aberrant NET formation[50]. Additionally, micronutrients such as zinc, selenium, and vitamin D are critical for the regulation of innate immunity and neutrophil-mediated responses. For instance, zinc plays a pivotal role in neutrophil chemotaxis and the generation of ROS in a controlled manner, while selenium helps mitigate oxidative damage and modulates inflammatory cytokine production. Vitamin D, beyond its classical roles, has been implicated in supporting antimicrobial peptide production and regulating neutrophil lifespan. These nutrients may act synergistically with antiretroviral therapy (ART), offering adjunctive benefits by restoring immune balance and potentially reducing HIV-associated comorbidities such as cardiovascular disease and neuroinflammation[51]. While these complementary approaches do not replace ART, their targeted integration into HIV care could offer a low-risk, supportive strategy to preserve neutrophil function and attenuate chronic inflammation. However, standardized clinical trials are needed to determine optimal dosing, timing, and combinations, ensuring safety and efficacy in diverse HIV-infected populations[52].

## Pitfalls of neutrophil-targeted therapies and their integration with antiretroviral therapy (ART)

As interest grows in targeting neutrophil dysfunction to manage HIV-associated inflammation and immune dysregulation, it is equally important to acknowledge the potential risks and complexities associated with such interventions. While neutrophils are central contributors to HIV-driven pathology through mechanisms like NET formation, excessive ROS production, and chronic activation, they also serve as frontline defenders against bacterial and fungal infections. Therefore, therapeutic strategies that impair neutrophil function carry the inherent risk of weakening innate immunity, which could heighten susceptibility to opportunistic infections, especially in immunocompromised individuals or those with advanced HIV[48]. For instance, inhibiting NETosis with agents like DNase I or blocking chemotactic receptors such as CCR5 may dampen unwanted inflammation, but they could also impair the neutrophil’s ability to localize and contain microbial threats, increasing the risk of sepsis or invasive infections. Similarly, broad suppression of neutrophil activation may result in unintended immunosuppression, potentially affecting the delicate balance required for host defense and immune recovery under ART[49]. Another concern is that neutrophils exhibit significant heterogeneity and plasticity in response to different tissue microenvironments and stages of HIV disease. Therapeutic targeting that does not account for this diversity may disrupt beneficial neutrophil subsets or inadvertently promote compensatory pathways, leading to unpredictable outcomes. Moreover, neutrophil-targeting interventions must be approached cautiously in individuals co-infected with pathogens like tuberculosis, where granulocytic responses are critical to containment[50].

Despite these pitfalls, a carefully integrated approach with existing ART regimens offers a promising solution. ART effectively suppresses viral replication and promotes partial immune reconstitution, but it does not fully reverse chronic inflammation or restore neutrophil homeostasis. Here, adjunctive therapies such as statins (e.g. atorvastatin) provide a strategic advantage. Statins exhibit anti-inflammatory properties without profoundly impairing antimicrobial responses. They can reduce endothelial activation, oxidative stress, and cytokine production, thereby modulating neutrophil behavior without directly disabling their defensive capacity^[51,52]^. Furthermore, agents like CCR5 inhibitors that serve dual roles – blocking viral entry and modulating immune cell trafficking – can be seamlessly incorporated into ART protocols, enhancing both antiviral and anti-inflammatory effects. The key lies in dose optimization, timing, and patient selection, ensuring that immune support is maintained while pathological activation is curtailed^[53,54]^.

## Clinical implications

Understanding the dualistic nature of neutrophil activity in HIV has significant clinical implications, particularly in the context of disease progression, comorbidity risk, and therapeutic management. On one hand, neutrophils contribute to frontline defense through mechanisms such as phagocytosis and the release of NETs, which help limit opportunistic infections common in immunocompromised individuals. On the other hand, persistent neutrophil activation can exacerbate systemic inflammation, contributing to non-AIDS-defining conditions such as cardiovascular disease, neurocognitive disorders, and metabolic syndrome. These complications, increasingly observed in people living with HIV despite effective ART, highlight the need to address inflammation beyond viral suppression^[55,56]^. Neutrophil dysfunction, including reduced antimicrobial responses and increased NETosis, may also impair mucosal barriers, further facilitating microbial translocation and chronic immune activation. Clinically, this necessitates monitoring not only viral load and CD4+ T cell counts but also inflammatory markers and neutrophil profiles. Moreover, therapeutic strategies targeting neutrophil activity – such as the use of statins, antioxidants, or chemokine receptor antagonists – must be carefully balanced to avoid compromising the host’s antibacterial defense. Integration of neutrophil-modulating therapies with ART could help reduce inflammatory burden and improve long-term outcomes. Thus, a deeper understanding of neutrophil behavior offers a promising avenue for more comprehensive HIV care, particularly in managing inflammation-driven comorbidities^[57–59]^.

## Future directions and recommendations

Future directions in understanding the role of neutrophils in HIV immunity should focus on addressing emerging and unresolved questions, particularly concerning the behavior of neutrophil subsets across different stages of HIV infection. One key area of interest lies in exploring how neutrophil phenotypes and functions differ during acute versus chronic HIV infection, as early immune responses may significantly influence disease progression and therapeutic outcomes. The identification and characterization of distinct neutrophil subsets – such as pro-inflammatory, immunosuppressive (e.g. G-MDSCs), or those involved in tissue repair – could clarify their specific contributions to HIV pathogenesis, immune activation, or viral dissemination^[60–62]^.

Advancements in technologies such as single-cell RNA sequencing and epigenetic profiling offer promising tools to unravel the heterogeneity of neutrophils at unprecedented resolution. These techniques can help delineate unique gene expression signatures, uncover activation states, and reveal functional plasticity in neutrophil populations that are otherwise masked in bulk analyses. Moreover, epigenetic studies may provide insights into how chronic HIV infection and inflammation reprogram neutrophil responses, potentially contributing to immune dysfunction and comorbidities. Applying these approaches in both experimental models and clinical cohorts will be essential to bridge existing knowledge gaps, identify novel biomarkers of disease progression, and refine targeted therapeutic strategies that preserve neutrophil function while minimizing immune pathology in HIV-infected individuals^[63–64]^.

## Conclusion

Neutrophils, traditionally recognized for their role in innate immunity against bacterial and fungal pathogens, exhibit dynamic interactions with HIV that influence both viral control and disease progression. On one hand, they contribute to antiviral defense through mechanisms such as the production of ROS, release of neutrophil extracellular traps (NETs), and modulation of adaptive immune responses. These mechanisms are essential for limiting viral replication and dissemination, potentially influencing the clinical course of HIV infection. On the other hand, HIV can induce dysfunction in neutrophils, compromising their ability to effectively combat infections and contributing to chronic inflammation, which is a hallmark of HIV pathogenesis. Chronic inflammation driven by neutrophils exacerbates tissue damage and contributes to the development of these comorbidities, emphasizing the broader impact of neutrophil-HIV interactions beyond viral control. Therapeutically, targeting neutrophil function and the inflammatory responses they mediate presents potential opportunities for enhancing HIV management strategies. Strategies aimed at modulating neutrophil activity, reducing chronic inflammation, and restoring immune balance could complement existing antiretroviral therapies and improve clinical outcomes for HIV-infected individuals.

## Data Availability

Not applicable as this is a narrative review.

## Research registration unique identifying number (UIN)

Not applicable as this a narrative review.

## References

[1] Obeagu EI Alum EU Obeagu GU. Factors associated with prevalence of HIV among youths: a review of Africa perspective. Madonna Univ J Med Health Sci 2023;3:13–18.

[2] Obeagu EI. A review of challenges and coping strategies faced by HIV/AIDS discordant couples. Madonna University Journal of Medicine and Health Sciences 2023;3:7–12.

[3] Obeagu EI Obeagu GU Musiimenta E. Factors contributing to low utilization of HIV counseling and testing services. Int J Curr Res Med Sci 2023;9:1–5.

[4] Rosales C. Neutrophils at the crossroads of innate and adaptive immunity. J Leucocyte Biol 2020;108:377–96. 10.1002/JLB.4MIR0220-574RR 32202340

[5] Smith JA. Neutrophils, host defense, and inflammation: a double‐edged sword. J Leukoc Biol 1994;56:672–86. 7996043 10.1002/jlb.56.6.672

[6] Obeagu EI Scott GY Amekpor F. Implications of CD4/CD8 ratios in human immunodeficiency virus infections. Int J Curr Res Med Sci 2023;9:6–13.

[7] Mehraj V Jenabian MA Vyboh K. Immune suppression by myeloid cells in HIV infection: new targets for immunotherapy. Open AIDS J 2014;8:66. 25624956 10.2174/1874613601408010066 PMC4302459

[8] Yaseen MM Abuharfeil NM Yaseen MM. The role of polymorphonuclear neutrophils during HIV-1 infection. Arch Virol 2018;163:1–21. 28980078 10.1007/s00705-017-3569-9

[9] Buckner LR Amedee AM Albritton HL. Chlamydia trachomatis infection of endocervical epithelial cells enhances early HIV transmission events. PloS One 2016;11:e0146663.10. 26730599 10.1371/journal.pone.0146663 PMC4701475

[10] Paiardini M Müller‐Trutwin M. HIV‐associated chronic immune activation. Immunol Rev 2013;254:78–101. 23772616 10.1111/imr.12079 PMC3729961

[11] Kolaczkowska E Kubes P. Neutrophil recruitment and function in health and inflammation. Nat Rev Immunol 2013;13:159–75. 23435331 10.1038/nri3399

[12] Kaltenmeier C Yazdani HO Morder K. Neutrophil extracellular traps promote T cell exhaustion in the tumor microenvironment. Front Immunol 2021;12:785222. 34899751 10.3389/fimmu.2021.785222 PMC8652262

[13] Okoye AA Picker LJ. CD 4+ T‐cell depletion in HIV infection: mechanisms of immunological failure. Immunol Rev 2013;254:54–64. 23772614 10.1111/imr.12066 PMC3729334

[14] Espíndola MS Soares LS Galvao-Lima LJ. HIV infection: focus on the innate immune cells. Immunol Res 2016;64:1118–32. 27590022 10.1007/s12026-016-8862-2

[15] Jiang AP Jiang JF Guo MG. Human blood-circulating basophils capture HIV-1 and mediate viral trans-infection of CD4+ T cells. J Virol 2015;89:8050–62. 26018157 10.1128/JVI.01021-15 PMC4505656

[16] Madzime M Rossouw TM Theron AJ. Interactions of HIV and antiretroviral therapy with neutrophils and platelets. Front Immunol 2021;12:634386. 33777022 10.3389/fimmu.2021.634386 PMC7994251

[17] Moodley M Moodley J Naicker T. The role of neutrophils and their extracellular traps in the synergy of pre-eclampsia and HIV infection. Curr Hypertens Rep 2020;22:1–9. 32462480 10.1007/s11906-020-01047-z

[18] Hensley-McBain T Wu MC Manuzak JA. Increased mucosal neutrophil survival is associated with altered microbiota in HIV infection. PLoS Pathog 2019;15:e1007672. 30973942 10.1371/journal.ppat.1007672 PMC6459500

[19] Doitsh G Greene WC. Dissecting how CD4 T cells are lost during HIV infection. Cell Host Microbe 2016;19:280–91. 26962940 10.1016/j.chom.2016.02.012 PMC4835240

[20] Johansson C Kirsebom FC. Neutrophils in respiratory viral infections. Mucosal Immunol 2021;14:815–27. 33758367 10.1038/s41385-021-00397-4 PMC7985581

[21] Britschgi M Pichler WJ. Acute generalized exanthematous pustulosis, a clue to neutrophil- mediated inflammatory processes orchestrated by T cells. Curr Opin Allergy Clin Immunol 2002;2:325–31. 12130947 10.1097/00130832-200208000-00006

[22] Casulli S Elbim C. Interactions between human immunodeficiency virus type 1 and polymorphonuclear neutrophils. J Innate Immun 2014;6:13–20. 23867213 10.1159/000353588 PMC6741617

[23] Peters-Golden M Canetti C Mancuso P. Leukotrienes: underappreciated mediators of innate immune responses. J Immunol 2005;174:58928. 10.4049/jimmunol.174.2.589 15634873

[24] Muralidharan A Reid SP. Complex roles of neutrophils during arboviral infections. Cells 2021;10:1324. 34073501 10.3390/cells10061324 PMC8227388

[25] Levy JA. Pathogenesis of human immunodeficiency virus infection. Microbiol Rev 1993;57:183–289. 8464405 10.1128/mr.57.1.183-289.1993 PMC372905

[26] Gonzalez SM Aguilar-Jimenez W Su RC. Mucosa: key interactions determining sexual transmission of the HIV infection. Front Immunol 2019;10:144. 30787929 10.3389/fimmu.2019.00144 PMC6373783

[27] Szabady RL McCormick BA. Control of neutrophil inflammation at mucosal surfaces by secreted epithelial products. Front Immunol 2013;4:220. 23914188 10.3389/fimmu.2013.00220 PMC3728559

[28] Day CJ Hardison RL Spillings BL. Complement receptor 3 mediates HIV-1 transcytosis across an intact cervical epithelial cell barrier: new insight into HIV transmission in women. Mbio 2022;13:e02177–21. 35012346 10.1128/mbio.02177-21 PMC8749410

[29] Neutra MR Pringault E Kraehenbuhl JP. Antigen sampling across epithelial barriers and induction of mucosal immune responses. Annu Rev Immunol 1996;14:275–300. 8717516 10.1146/annurev.immunol.14.1.275

[30] Hiroki CH Toller-Kawahisa JE Fumagalli MJ. Neutrophil extracellular traps effectively control acute chikungunya virus infection. Front Immunol 2020;10:3108. 32082301 10.3389/fimmu.2019.03108 PMC7005923

[31] Lamichhane PP Samarasinghe AE. The role of innate leukocytes during influenza virus infection. J Immunol Res 2019;2019:1–17. 10.1155/2019/8028725 PMC6757286 31612153

[32] Hensley-McBain T Klatt NR. The dual role of neutrophils in HIV infection. Curr HIV/AIDS Rep 2018;15:1–0. 29516266 10.1007/s11904-018-0370-7 PMC6086572

[33] Kumar V Sharma A. Neutrophils: Cinderella of innate immune system. Int Immunopharmacol 2010;10:1325–34. 20828640 10.1016/j.intimp.2010.08.012

[34] Mócsai A. Diverse novel functions of neutrophils in immunity, inflammation, and beyond. J Exp Med 2013;210:1283–99. 23825232 10.1084/jem.20122220 PMC3698517

[35] Cribbs SK Crothers K Morris A. Pathogenesis of HIV-related lung disease: immunity, infection, and inflammation. Physiol Rev 2020;100:603–32. 31600121 10.1152/physrev.00039.2018

[36] Kobayashi SD Voyich JM Burlak C. Neutrophils in the innate immune response. Arch Immunologiae Et Ther Exp-Engl Ed 2005;53:505. 16407783

[37] Reno TA Tarnus L Tracy R. The youngbloods. Get together. Hypercoagulation, complement, and NET formation in HIV/SIV pathogenesis. Front Virol 2022;1:795373.

[38] Lv T Cao W Li T. HIV-related immune activation and inflammation: current understanding and strategies. J Immunol Res 2021;2021:1–13. 10.1155/2021/7316456 PMC8494587 34631899

[39] Boasso A Shearer GM Chougnet C. Immune dysregulation in human immunodeficiency virus infection: know it, fix it, prevent it? J Intern Med 2009;265:78–96. 19093962 10.1111/j.1365-2796.2008.02043.x PMC2903738

[40] Freeman ML Shive CL Nguyen TP. Cytokines and T-cell homeostasis in HIV infection. J Infect Dis 2016;214:S51–7. 27625431 10.1093/infdis/jiw287 PMC6373575

[41] Paiardini M Müller‐Trutwin M. HIV‐associated chronic immune activation. Immunol Rev 2013;254:7847. 10.1111/imr.12079 PMC3729961 23772616

[42] Leliefeld PH Koenderman L Pillay J. How neutrophils shape adaptive immune responses. Front Immunol 2015;6:471. 26441976 10.3389/fimmu.2015.00471 PMC4568410

[43] Hahn S Giaglis S Chowdury CS. Modulation of neutrophil NETosis: interplay between infectious agents and underlying host physiology. InSeminars Immunopathol 2013;35:439–53. 10.1007/s00281-013-0380-x PMC3685704 23649713

[44] Futosi K Fodor S Mócsai A. Neutrophil cell surface receptors and their intracellular signal transduction pathways. Int Immunopharmacol 2013;17:638–50. 23994464 10.1016/j.intimp.2013.06.034 PMC3827506

[45] George ST Lai J Ma J. Neutrophils and influenza: a thin line between helpful and harmful. Vaccines (Basel) 2021;9:597. 34199803 10.3390/vaccines9060597 PMC8228962

[46] Klatt NR Chomont N Douek DC. Immune activation and HIV persistence: implications for curative approaches to HIV infection. Immunol Rev 2013;254:326–42. 23772629 10.1111/imr.12065 PMC3694608

[47] Mojoli A Gonçalves BS Temerozo JR. Neutrophil extracellular traps from healthy donors and HIV-1-infected individuals restrict HIV-1 production in macrophages. Sci Rep 2020;10:19603. 33177532 10.1038/s41598-020-75357-2 PMC7658358

[48] Özcan A Boyman O. Mechanisms regulating neutrophil responses in immunity, allergy, and autoimmunity. Allergy 2022;77:3567–83. 36067034 10.1111/all.15505 PMC10087481

[49] Govender NP Meintjes G Mangena P. Southern African HIV Clinicians Society guideline for the prevention, diagnosis and management of cryptococcal disease among HIV-infected persons: 2019 update. South Afr J HIV Med 2019;20:1–6. 10.4102/sajhivmed.v20i1.1030 PMC7081625 32201629

[50] Mantovani A Cassatella MA Costantini C. Neutrophils in the activation and regulation of innate and adaptive immunity. Nat Rev Immunol 2011;11:519–31. 21785456 10.1038/nri3024

[51] Clarke JO Mullin GE. A review of complementary and alternative approaches to immunomodulation. Nutrition in Clinical Practice 2008;23:49–62. 18203964 10.1177/011542650802300149

[52] Titanji B Gavegnano C Hsue P. Targeting inflammation to reduce atherosclerotic cardiovascular risk in people with HIV infection. J Am Heart Assoc 2020;9:e014873. 31973607 10.1161/JAHA.119.014873 PMC7033865

[53] Obeagu EI Obeagu GU Ukibe NR. Anemia, iron, and HIV: decoding the interconnected pathways: a review. Medicine (Baltimore) 2024;103:e36937. 38215133 10.1097/MD.0000000000036937 PMC10783375

[54] Alum EU Obeagu EI Ugwu OPC. Inclusion of nutritional counseling and mental health services in HIV/AIDS management: a paradigm shift. Medicine (Baltimore) 2023;102:e35673. 37832059 10.1097/MD.0000000000035673 PMC10578718

[55] Obeagu EI. Diagnostic and prognostic significance of mast cell markers in HIV/AIDS: current insights and future directions. Medicine (Baltimore) 2024;103:e38117. 38758896 10.1097/MD.0000000000038117 PMC11098248

[56] Anyiam AF Arinze-Anyiam OC Irondi EA. Distribution of ABO and rhesus blood grouping with HIV infection among blood donors in Ekiti State Nigeria. Medicine (Baltimore) 2023;102:e36342. 38013335 10.1097/MD.0000000000036342 PMC10681551

[57] Aizaz M Abbas FA Abbas A. Alarming rise in HIV cases in Pakistan: challenges and future recommendations at hand. Health Sci Rep 2023;6:e1450. 37520460 10.1002/hsr2.1450 PMC10375546

[58] Opeyemi AA Obeagu EI. Regulations of malaria in children with human immunodeficiency virus infection: a review. Medicine (Baltimore) 2023;102:e36166. 37986340 10.1097/MD.0000000000036166 PMC10659731

[59] Obeagu EI Obeagu GU Ede MO. Translation of HIV/AIDS knowledge into behavior change among secondary school adolescents in Uganda: a review. Medicine (Baltimore) 2023;102:e36599. 38065920 10.1097/MD.0000000000036599 PMC10713174

[60] Obeagu EI Obeagu GU. Platelet index ratios in HIV: emerging biomarkers for immune health and disease management. Medicine (Baltimore) 2024;103:e37576. 38518025 10.1097/MD.0000000000037576 PMC10956946

[61] Obeagu EI Obeagu GU. Utilization of immunological ratios in HIV: implications for monitoring and therapeutic strategies. Medicine (Baltimore) 2024;103:e37354. 38428854 10.1097/MD.0000000000037354 PMC10906605

[62] Echefu SN Udosen JE Akwiwu EC. Effect of Dolutegravir regimen against other regimens on some hematological parameters, CD4 count and viral load of people living with HIV infection in South Eastern Nigeria. Medicine (Baltimore) 2023;102:e35910. 38013350 10.1097/MD.0000000000035910 PMC10681510

[63] Xu H Wang X Veazey RS. Mucosal immunology of HIV infection. Immunol Rev 2013;254:10–33. 23772612 10.1111/imr.12072 PMC3693769

[64] Maslin CL Kedzierska K Webster NL. Transendothelial migration of monocytes: the underlying molecular mechanisms and consequences of HIV- 1 infection. Curr HIV Res 2005;3:303–17. 16250878 10.2174/157016205774370401

